# Facile synthesis of Au/ZnO/Ag nanoparticles using *Glechoma hederacea L.* extract, and their activity against leukemia

**DOI:** 10.1007/s10544-021-00557-0

**Published:** 2021-03-08

**Authors:** Renata Dobrucka, Aleksandra Romaniuk-Drapała, Mariusz Kaczmarek

**Affiliations:** 1 Department of Non-Food Products Quality and Packaging Development, Institute of Quality Science, Poznań University of Economics and Business, al. Niepodległości 10, 61-875 Poznań, Poland; 2 Department of Clinical Chemistry and Molecular Diagnostics, Poznan University of Medical Sciences, 49 Przybyszewskiego St, 60-355 Poznań, Poland; 3 Department of Cancer Immunology, Chair of Medical Biotechnology, Poznan University of Medical Sciences, Garbary 15 Str, 61-866 Poznan, Poland; 4 Gene Therapy Laboratory, Department of Cancer Diagnostics and Immunology, Greater Poland Cancer Centre, Garbary 15 Str, 61-866 Poznan, Poland

**Keywords:** Synthesis, Au/ZnO/Ag nanoparticles, *Glechoma hederacea L.*, Leukemia, Cancer

## Abstract

Metal combinations have been attracting the attention of scientists for some time. They usually exhibit new characteristics that are different from the ones possessed by their components. In this work, Au/ZnO/Ag nanoparticles were synthesized biologically using *Glechoma hederacea* L. extract. The synthesized Au/ZnO/Ag nanoparticles were characterized by UV-Vis, Scanning electron microscopy (SEM), Fourier transform infrared spectroscopy (FTIR), Transmission electron microscopy (TEM), and Atomic Force Microscopy (AFM). The microscopic methods confirmed the presence of spherical nanoparticles of 50–70 nm. The influence of biologically synthesized Au/ZnO/Ag nanoparticles on the vitality of human cells was evaluated *in vitro* with the use of established human Acute T Cell Leukemia cell line, Jurkat (ATCC® TIB-152™), as well as mononuclear cells isolated from peripheral blood (PBMC) of voluntary donors. Cell survival and the half-maximal inhibitory concentration index (IC50) were analyzed by the MTT test. The studies showed that the total loss of cell viability occurred at the Au/ZnO/Ag nanoparticle concentration range of 10 µmol–50 µmol. The use of Au/ZnO/Ag nanoparticles at the concentration of 100 µmol eliminated almost all living cells from the culture in 24h. The above observation confirms the result obtained during the MTT test.

## Introduction

Conventional diagnostic approaches to cancer have certain limitations, such as inability to detect carcinoma at the primary stage, inability to differentiate between malignant or benign tumors, high false positive signals etc. [1]. Nanotechnology is a multidisciplinary science based on concepts of chemistry, biochemistry, physics, and materials science, which has enormous utility in medicine [2]. Nanosized materials have been an important subject in basic and applied science. For many years, special attention has been devoted to metal nanoparticles. Numerous studies show that metal nanoparticles can easily be transported in the body, they can penetrate cell membranes and accumulate in target sites. In addition, they can be retained in mitochondria and cause cytotoxic effects. The utilization of nanoparticles in treatment and management of diseases, including cancer, creates opportunities to potentially destroy cancer cells while minimizing damage to the cells and tissues that are healthy. The literature provides a broad account of how metal nanoparticles can effectively impact numerous types of cancer, such as MCF-7 breast cancer cell lines [3], He-La Cervical Cancer Cell Lines [4], hepatocellular carcinoma (HepG2) [5], skin cancer [6] and ovarian cancer [7].

Cancer is a complex disease which develops in cells of specific tissue that are no longer receptive to signals regulating differentiation of cells, survival, proliferation and death [8]. Leukemia is considered to be a multistep progression by progressive genetic modifications that cause normal human hematopoietic progenitor/stem cells to convert into leukemic cells. Leukemia originates from a single cell that has been subjected to malignant conversion by way of regular genetic mutations [9]. At the early stage, it prolifetares at a slow pace in immature white blood cells, bone marrow, and the lymphoid system. Next, there is a rapid spread to lymph nodes, spleen, liver and central nervous system (brain and spinal cord). Several therapeutic methods are used to treat leukemia. These methods include stem cell transplantation, tyrosine kinase inhibitors for positive leukemia, A-CD22 directed therapy, epratumab, obinutuzumab, REGN1979, C-CD19, Coltuximab ravtansine, SGN-CD 19A, ADCT-402, D-CD25, ADCT-301, Inotuzumab ozogamicin, moxetumomab pasudodotox, combotox, B-CD20, Ofatumumab, Bortezomib, Ruxolitinib, Decitabine, P13K/mTOR inhibitor, 6-chimeric antigen receptor T cells, and rituximab [10]. As the understanding of biomedical characteristics of nanoparticles is getting better, we observe a continuous development of cancer nanotechnology. The last few years have brought an extraordinary evolution as regards the design and use of many nanomaterials in cancer nanotechnology [11]. However, the majority of those studies focus on the activity of monometallic nanoparticles, whereas recent years have seen a growing interest in metal combinations, including bi- and tri-nanometals. Metal nanoparticle combinations are causing a greater research awareness due to the fact that they exhibit new chemical and physical properties, which is a result of synergy between their individual components [12].

One of important aspects of nanotechnology concerns the process of synthesizing nanoparticles. Various methods for synthesizing metal nanoparticles include physical vapor deposition, chemical vapor deposition, sol–gel method, microwave-assisted synthesis, ultrasonication method, electrochemical synthesis, and chemical reduction of metallic ions [13]. For several years, metallic nanoparticles have been produced using low-cost, energy-efficient and non-toxic methods utilizing plants, algae, fungi, bacteria and viruses [14]. The application of phytochemicals for the purpose of synthesizing nanoparticles represents a significant symbiosis between nanotechnology and green chemistry [15]. The advantages of green synthesis of metal nanoparticles include greater biocompatibility, convenient scale-up, straightforward procedures of reactions, and more [16]. In this work, we synthesized Au/ZnO/Ag nanoparticles using *G. hederacea* L. extract. *G. hederacea* L. (*Lamiaceae*), commonly known as ground ivy, is a perennial plant widely distributed in Europe, Asia and America [17]. *G. hederacea* L. belongs to the Labiatae family. It contains essential oil (rich in camphene, pinocamphene, pinene, myrcene, menthone, pulegone), triterpenes and sesquiterpenes (glechomanolid, glechomafuran, ursolic acid, oleanolic acid and their derivatives), tannins (6–7%), flavonoids (quercetin, luteolin, apigenin), phenolic acids (rosmarinic, ferulic, caffeic, syringic, vanillic acid), bitters, and saponin glycosides. Due to the presence of such a high number of bioactive compounds acting as reducing and capping agents, *G.hederacea* L. was used in this work to synthesize Au/ZnO/Ag nanoparticles.

## Materials and methods

### Synthesis of Au/ZnO/Ag nanoparticles

The synthesis of Au/ZnO/Ag nanoparticles started with preparation of the *Glechoma hederacea* L. extract, which was made by combining 10 g of powdered *G. hederacea L* and 100 ml of double distilled water. The prepared solution was subjected to heating and vigorous stirring for 45 minutes at 60°C. Then, 3 solutions were prepared: 5 mM AgNO_3_, 5 mM HAuCl_4_, and 5 mM ZnNO_3_, and they were combined at a 1:1:1 ratio. Prior to absorbance readings, the solution was stirred for 12h at 60°C [18].

### Characterization of Au/ZnO/Ag nanoparticles

The maximum absorbance of the sample was measured by UV-Visible spectrophotometry. Ultraviolet and visible absorption spectroscopy (spectrophotometer Cary E 500) at the range of 300 nm–600 nm were used to analyze the optical properties of Au/ZnO/Ag nanoparticles synthesized using *G. hederacea* L. extract. Fourier transform infrared spectroscopy (FTIR) analysis was used to determine the binding properties of Au/ZnO/Ag nanoparticles synthesized with the use of *G. hederacea* L. extract. FTIR was also used to characterize Au/ZnO/Ag nanoparticles synthesized with the use of *G. hederacea* L. extract. The shape, size and microstructures of Au/ZnO/Ag nanoparticles synthesized using *G. hederacea* L. extract were determined with a Transmission electron microscope JEOL JEM 1200 EXII operating at 80 kV. TEM involves a beam of electrons that passes through, and interacts with, an extremely thin specimen. When accelerated to a high extent (up to several hundreds of keV) and focused on a given material, electrons can scatter or backscatter, elastically or inelastically, as well as interact in many ways and emit various signals, e.g. X-rays, Auger electrons or light [19]. The study was conducted using the atomic force microscope INTEGRA SPECTRA SOLAR of NT-MDT brand and measurement tips dedicated for NSGO1 high-resolution measurements, in the tapping mode. The resonance frequency of the tips ranged from 87 to 230 kHz. The force constant ranged from 1.45 to 15.1 N/m. The scanning area was 10 µm x 10 µm, with 1000 x 1000 scanning points. The picture of Au/ZnO/Ag nanoparticles synthesized using *G. hederacea* L. extract was obtained by means of Scanning electron microscopy (SU3500), Hitachi.

### Evaluation of cytotoxic activity of Au/ZnO/Ag nanoparticles

The effects of Au/ZnO/Ag nanoparticles on human cell vitality were examined *in vitro* using the established human Acute T Cell Leukemia cell line, Jurkat (ATCC® TIB-152™), and mononuclear cells that were isolated from peripheral blood (PBMC) obtained from voluntary donors. PBMC contains peripheral blood lymphocytes (PBL) and monocytes. Cell cultures were carried out in suspension in RPMI 1640 medium with 2mM L-glutamine with the addition of 10% fetal bovine serum (FBS) and 1% Gibco® Antibiotic-Antimycotic solution (10,000 units/mL of penicillin, 10,000 µg/mL of streptomycin, and 25 µg/mL of Amphotericin B) on 24-well plastic plates (TC-PLATE 24 well, Greiner). Cells were cultured in an incubator in a 5% CO_2_ atmosphere with increased humidity, at 37°C. Au/ZnO/Ag *nanoparticles* were added at three different concentrations: 1μM, 10μM, and 100μM. Samples without the addition of Au/ZnO/Ag nanoparticles served as controls. All the tested samples, both Jurkat cells, and PBMC were cultured for the period of 24, 48 and 72 hours. All determinations were made in triplicates during two independent tests.

### Separation of PBMC

Whole samples of peripheral blood were collected from voluntary donors in heparin tubes. Next, blood was mixed with sterile Phosphate-Buffered Saline (PBS) at a 1:1 ratio, and placed on the upper layer of Histopaque®-1077 solution (Sigma-Aldrich). The PBMC separation procedure was performed according to the protocol attached to the commercially available kit. Histopaque®-1077 is a polysucrose and sodium diatrizoate solution, whose density is 1.077 g/mL. It enables the separation of viable lymphocytes and other mononuclear cells from small amounts of whole blood. After centrifugation, which was carried out for 20 minutes at 1500 rpm in RT, the buffy coat layer with mononuclear cells was aspirated and placed on 24-well culture plates.

### MTT test (cell viability assay)

The MTT test was used to analyze cell survival and the half-maximal inhibitory concentration index (IC50). The IC50 value shows the concentration of tested substances which is needed to inhibit, *in vitro*, the biological activity of cells by 50%. The cytotoxicity level was established by estimating the percentage of dead cells and the degree of growth inhibition. The MTT test was performed according to the protocol provided with the reagent (Sigma-Aldrich). The MTT test measures the reduction of MTT tetrazolium salts soluble in water (3-(4,5-dimethylthiazol-2-yl)-2,5-diphenyltetrazolium bromide) to blue-violet insoluble formazan crystals by active mitochondrial dehydrogenase. This test makes it possible to carry out a quantitative assessment of cell proliferation by measuring the linear relationship between cell activity and the absorbance of color reaction [20]. Before the absorbance was read, formazan crystals were extracted from the cells and solubilized with 10% SDS in 0.01M HCl solution. The absorbance was measured with a microplate reader (Multiscan, Labsystems, Thermo Fisher Scientific Inc.) at the wavelenghts of 570/690 nm. Jurkat cells were placed on the culture wells in the amount of 5×10^3^ cells/well. Then, cells cultured with the addition of Au/ZnO/Ag *nanoparticles* in the above-described conditions were treated with 10μl of MTT solution (5 mg/ml thiazolyl blue Tetrazolium Bromide). Following 4 hours of incubation, formazan crystals were released from the cells using 100 μl of a solubilizing solution. The results of the assay were presented as Relative Viability of Cells (RVC) value, i.e. the ratio between the absorbance value for cells that were cultured in the presence of the tested nanoparticles, to the absorbance value for control samples [21, 22]. The RVC value was calculated according to the formula specified below proposed by us, in which (a) means the absorbance of the tested sample; (b) means the absorbance of the blank control (pure medium with no cells); and (c) means the absorbance of control cells with no addition of Au/ZnO/Ag nanoparticles.

$$\mathrm{RVC}(\mathrm{\%})=[(\mathrm{a}-\mathrm{b})/(\mathrm{c}-\mathrm{b})]\mathrm{x}100$$

### Evaluation of apoptosis with Annexin V

The influence of Au/ZnO/Ag nanoparticles on the viability of the Jurkat cell line was also assessed by taking into consideration the initiation of apoptosis or necrosis. The apoptotic or necrotic death was evaluated with a FITC Annexin V Apoptosis Detection Kit I (BD Pharmingen) available commercially, in accordance with the procedure specified by the manufacturer. Similarly as in the case of the MTT assay, cell cultures were carried out in the presence of Au/ZnO/Ag *nanoparticles* at the concentration of 1μM, 10μM, and 100μM for 24, 48, and 72h. The test was conducted in the following manner: first, cells were suspended in 100μl of Annexin buffer (1×), in the amount of 1×10^5^ cells. Then, 5μl of propidium iodide (PI) and 5μl of Annexin V conjugated with fluorescein (FITC) were added to the samples. After incubation in the dark for 15 minutes, 400μl of Annexin buffer (1×) were added to each of the test tubes. Stained samples were acquired with FACS Canto flow cytometer (Becton Dickinson). FACS Diva Software (Becton Dickinson) was used to analyze the results. Based on the proportion between FITC and/or PI fluorescence, cells were defined as early apoptotic, late apoptotic or necrotic [23]. Data were obtained after two independent measurements.

### Cell cycle evaluation

The evaluation of the proliferative activity of the Jurkat cell line and PBMC cells cultured in the presence of Au/ZnO/Ag nanoparticles was based on the cell cycle. For this purpose, a fluorescent dye intercalating into DNA structure, propidium iodide (PI), was used. In this test, the mean fluorescence intensity (MFI) emitted by PI, which intercalates into the DNA of replicating cells, serves as the basis for determining the percentage of cells. The MFI emitted by PI depends on the cell cycle phase. Proliferactive activity is measured on the basis of the percentage of cells in the S phase of cell cycle. The number of cells in the S phase of cell cycle makes it possible to determine the level of cells in the replication process. In addition, cytometric histograms make it possible to determine the percentage of cells before mitosis (G2/M phase) and the percentage of dead cells. For the purpose of the assay, cells were placed on culture plates in triplicates, 5×10^4^ cells per well, and cultured for 24, 48, and 72 hours in the presence of Au/ZnO/Ag nanoparticles at the following concentrations: 1μM, 10μM, and 100μM. Then, cells were prepared in the following manner: first they were permeabilized with BD Perm/Wash™ Buffer (BD Biosciences), and then they were stained with a 100μg/ml PI solution (Sigma-Aldrich). Both incubations were carried out in the fridge at 4℃ for 30 minutes, while ensuring protection from light. Finally, the FACS Canto flow cytometer (Becton Dickinson) was used to add the stained cells to the acquisition. Histograms were analyzed using FACS Diva software (Becton Dickinson) [24].

### Statistical analysis

The statistical analysis was performed using ANOVA.

## Results and discussion

### UV VISstudies of Au/ZnO/Ag nanoparticles

**Fig. 1:**
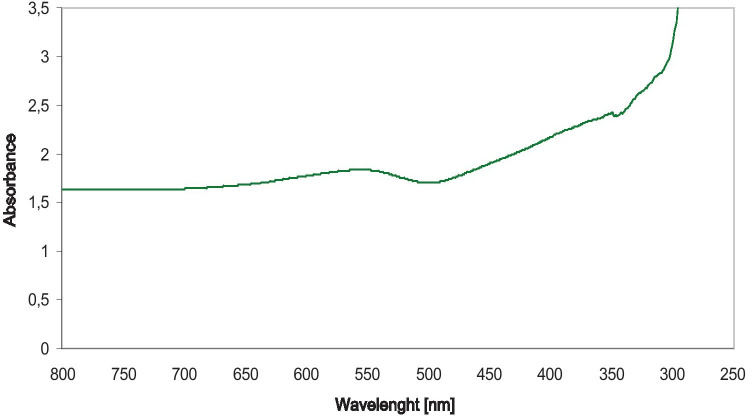
UV–visible spectra of Au/ZnO/Ag nanoparticles synthesized using of using *G. hederacea* L. extract

Figure 1 shows the UV–visible spectra of Au/ZnO/Ag nanoparticles synthesized from *G. hederacea* L. extract. UV–visible spectrophotometry is a suitable method for monitoring the progress of reactions or observing the surface plasmon resonance (SPR) of metallic nanoparticles [25]. In this study, the absorbance of the solution was measured in a wavelength range of 250–800 nm. The absorption spectra of the reaction media showed absorbance at 570 nm, confirming the presence of gold and silver nanoparticles. In addition, absorbance increase was observed at the wavelength of 352 nm, which is characteristic of ZnO nanoparticles. Moreover, a change in color visually confirmed the formation of trimetallic nanoparticles. After the extract was mixed with metal nanoparticle precursors, the solution rapidly changed its color from light beige to dark beige. Such a clear change of color confirms the reduction of metals to the nano form. Similar changes have been observed by Thiruchelvi et al. [26] and Adewale et al. [27]. These authors [27] synthesized AgNPs and AuNPs. According to the researchers colour changes suggest reduction and the synthesis of the nanoparticles, which are related to the surface plasmon resonance (SPR) band.

### Fourier transform infrared spectroscopy (FTIR) studies of Au/ZnO/Ag nanoparticles

**Fig. 2:**
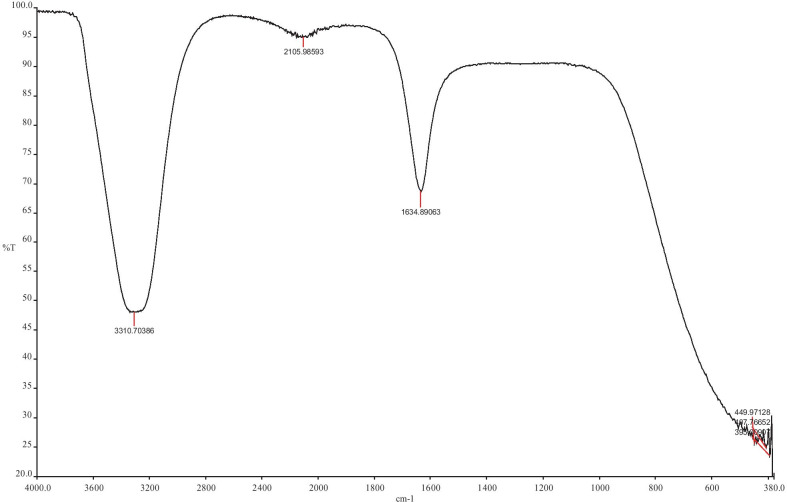
FTIR spectra of the Au/ZnO/Ag nanoparticles synthesized using of using *G. hederacea* L. extract

The FT-IR measurement, shown in Fig. 2, was conducted in the wave number range from 380 to 4000 cm−1, at room temperature, with the use of the KBr method. The strong peaks were observed at 3310 cm−1, 2105 cm−1, 1634 cm−1, 449 cm−1, 407 cm−1 and 395 cm−1. The strong absorption peak at 3310 cm−1 is related to -OH stretching and the aliphatic methylene group -C-H stretching. The most intense band at 1634 cm−1 represents C=O vibrations, which are typical of the structure of phenolic acids present in *Glechoma hederacea* L. The spectrum showed bands at 449 cm−1, 407 cm−1 and 395 cm−1 indicating the formation of metal-biomolecules present in the extract. Fourier transform infrared spectroscopy (FTIR) studies confirmed that *G. hederacea* L. contains bioactive compounds acting as reducing and capping agents for Au/ZnO/Ag nanoparticles.

**Fig. 3:**
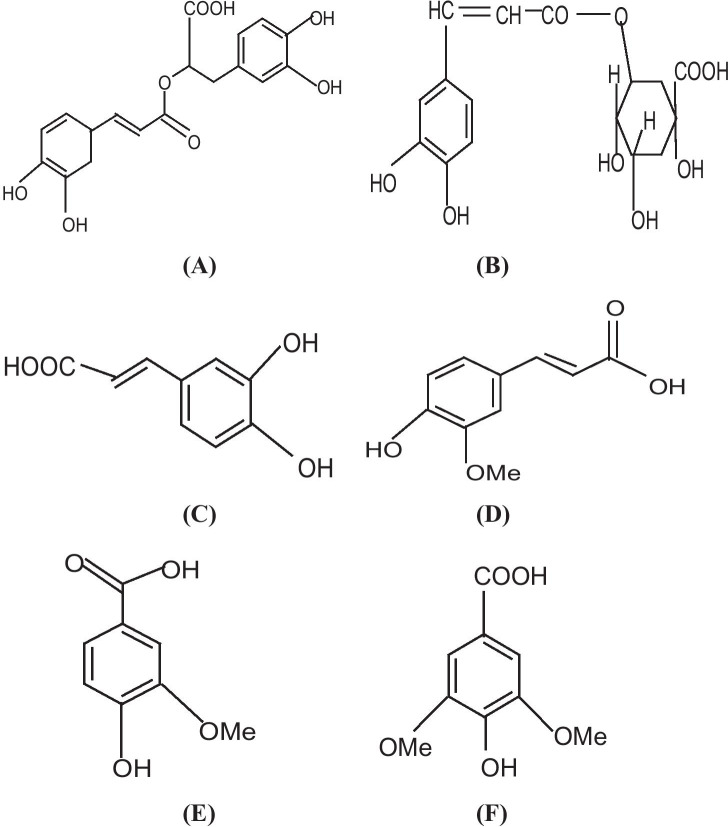
The chemical structure of bioactive compounds present in *G. hederacea* L. (**A**) rosmarinic acid, (**B**) chlorogenic acid, (**C**) caffeic acid, (**D**) ferulic acid, (**E**) vanillic acid, (**F**) syringic acid

According to the literature, *G. hederacea* L. contains such compounds as essential oil (rich in camphene, pinocamphene, pinene, myrcene, menthone, pulegone), triterpenes and sesquiterpenes (glechomanolid, glechomafuran, ursolic acid, oleanolic acid and their derivatives), tannins (6–7%), flavonoids (quercetin, luteolin, apigenin), phenolic acids (rosmarinic, ferulic, caffeic, syringic, vanillic, chlorogenic acid), bitters, and saponin glycosides. It also contains triterpenes, sesquiterpenes, phenolic acids and saponosides [28]. According to Khojasteh et. al. [29] rosmarinic acid and caffeic acid are common water-soluble phenolic compounds found mainly in plants of the Lamiaceae family. Chizzola et al. [30] reported that rosmarinic acid influenced 2,2-diphenyl-1-picrylhydrazyl (DPPH) activity. In particular, it was shown that the antiradical activity of rosmarinic acid and other caffeic acid derivatives was higher than that exerted by flavonoids [31]. Figure 3 shows the chemical structure of bioactive compounds present in *G. hederacea* L. (A) rosmarinic acid, (B) chlorogenic acid, (C) caffeic acid, (D) ferulic acid, (E) vanillic acid and (F) syringic acid.

Polyphenols, particularly flavonoids, are natural antioxidant substances with powerful Reactive Oxygen Species (ROS) scavenging properties that strongly reduce the risk of oxidative stress [32]. The presence of flavonoids is very significant for the formation Au/ZnO/Ag nanoparticles. The antioxidant activity of flavonoid compounds is associated with the ring-shaped structure of the particle, which contains conjugated double bonds, as well as with the presence of various functional groups in the rings [14]. Flavonoids exhibit antioxidant properties due a considerable number of hydroxyl groups. The intensity of their antioxidant activity depends on the number and position of hydroxyl groups. Compounds with a greater number of hydroxyl groups have stronger antioxidant properties. It was shown that myricetin (which has 6 hydroxyl groups) exhibits greater antioxidant properties than, for example, kaempferol, which has 4 hydroxyl groups. Among the hydroxyl groups present in a particle, the presence of two groups in ring B in the ortho position is particularly significant. Although it has been demonstrated that a particle which has hydroxyl groups in the para position exhibits a higher antioxidant activity than a structure with an ortho group, such a structure is present only in some flavonoids, which are rarely encountered. The presence of hydroxyl groups in the meta position does not affect the antioxidant properties of a compound [33].

### Atomic Force Microscopy (AFM) studies of Au/ZnO/Ag nanoparticles

**Fig. 4:**
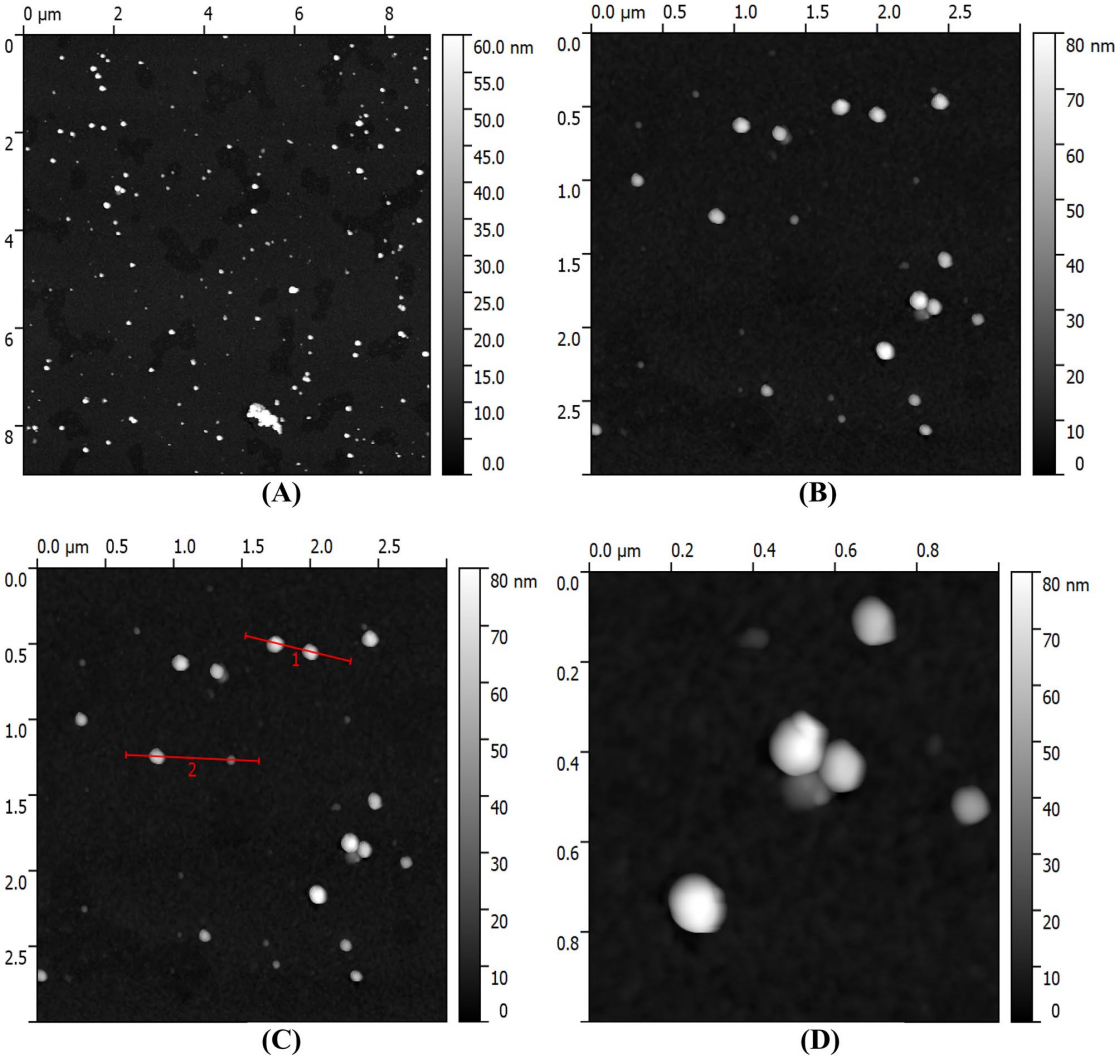
AFM images of Au/ZnO/Ag nanoparticles synthesized using of using *G.hederacea* L. extract with (**A**) the topography of 10 µm x 10 µm (**B**) the topography of 3 µm x 3 µm, (**C**) the topography of 3µm x 3µm with the profile and (**D**) the topography of 1 µm x 1 µm

The size of the Au/ZnO/Ag nanoparticles synthesized from *G. hederacea* L. was analyzed by means of Atomic Force Microscopy. Figure 4 presents the AFM images of Au/ZnO/Ag nanoparticles synthesized using *G. hederacea* L. extract with (A) the topography of 10 µm x 10 µm; (B) the topography of 3 µm x 3 µm; (C) the topography of 3µm x 3µm with the profile; and (D) the topography of 1 µm x 1 µm. The use of Atomic Force Microscopy made it possible to observe spherical particles of 50–70 nm [34].

### Scanning electron microscopy (SEM) studies of Au/ZnO/Ag nanoparticles

**Fig. 5:**
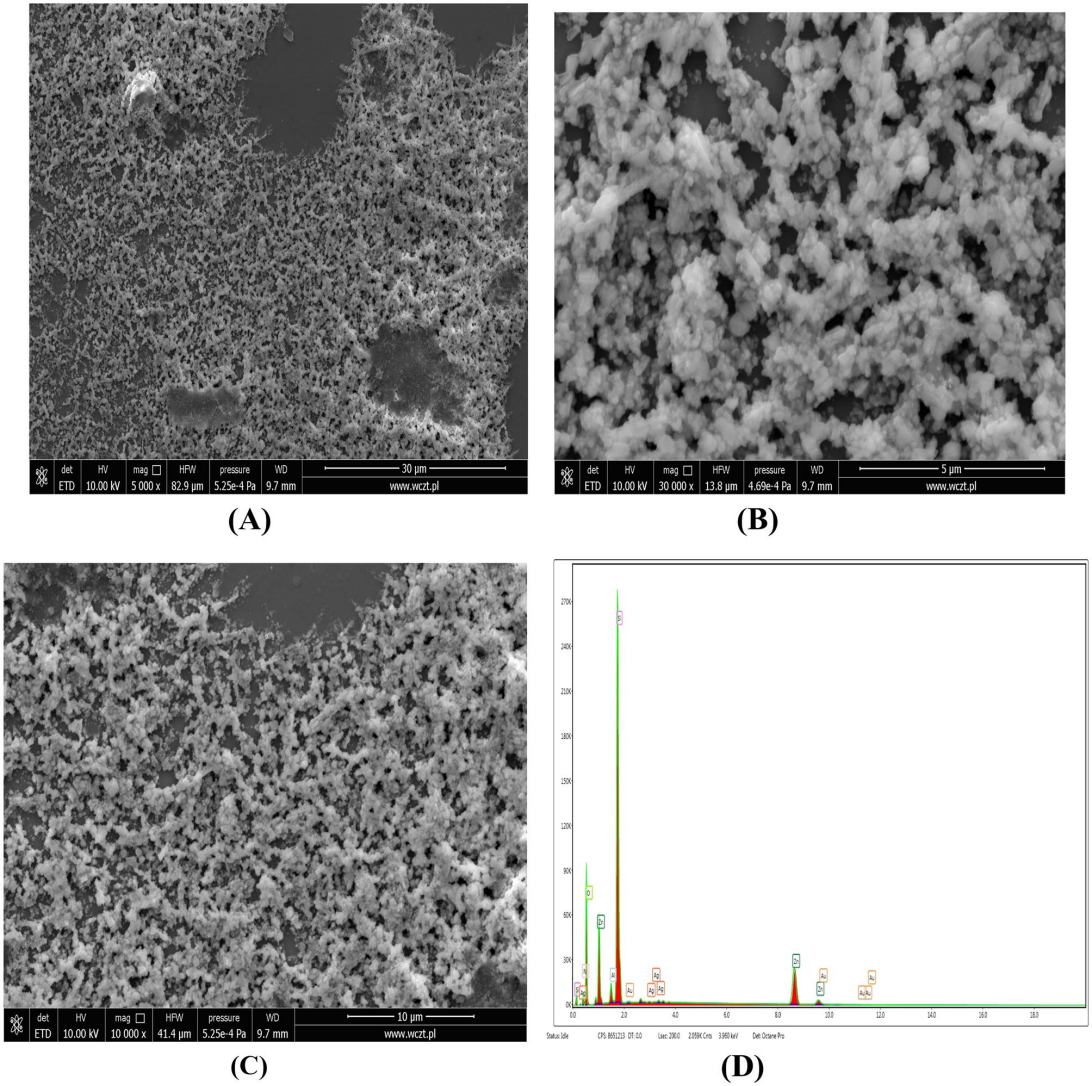
SEM images of Au/ZnO/Ag nanoparticles synthesized using of using *G.hederacea* L. extract at magnification (**A**) 5000X, (**B**) 30 000X, (**C**) 10000 and (**D**) EDS spectrum

The morphology of Au/ZnO/Ag nanoparticles synthesized using *G. hederacea* L. extract was observed by means of scanning electron microscopy (SEM). Figure 5 shows the SEM images of Au/ZnO/Ag nanoparticles synthesized using *G. hederacea* L. extract at the following magnifications: (A) 5000X, (B) 30 000X, (C) 10000 and (D) EDS spectrum. Figure 5 (D) presents EDS studies. In tested sample, the peaks were observed which confirmed the presence of three metals nanoparticles. Scanning microscopy made it possible to observe and confirm the presence of spherical particles that were locally agglomerated due to the presence of ZnO nanoparticles [35].

### Transmission Electron Microscope Analysis (TEM) studies of Au/ZnO/Ag nanoparticles

**Fig. 6:**
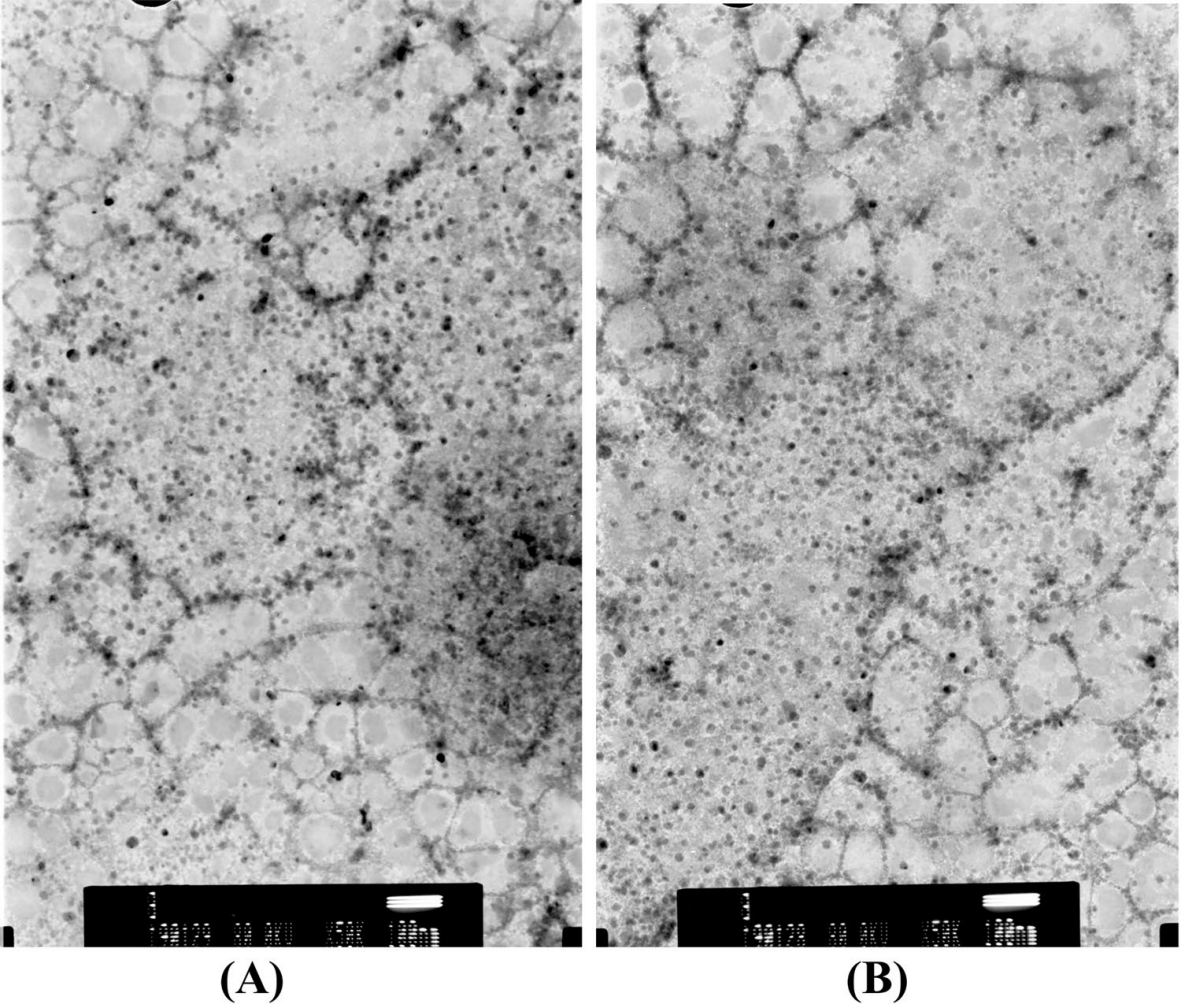
TEM image of Au/ZnO/Ag nanoparticles synthesized using of using *G.hederacea* L. extract with the scale bar (**A**) 200 nm and (**B**) 100 nm

The size and shape of Au/ZnO/Ag nanoparticles synthesized using *G.hederacea* L. were assessed by means of Transmission Electron Microscope (TEM) analysis [36]. The TEM images used in the work indicated the presence of Au/ZnO/Ag nanoparticles that were spherical in shape. Figure 6 presents the TEM image of Au/ZnO/Ag nanoparticles synthesized from *G. hederacea* L. extract with the scale bar of (A) 200 nm and (B) 100 nm.

### Cytotoxic activity of Au/ZnO/Ag nanoparticles

## MTT test

**Fig. 7:**
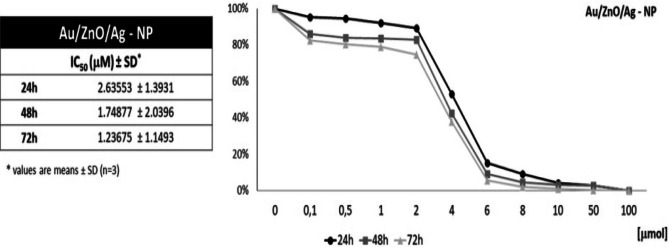
The Relative Cell Viability curve based on the MTT test for the Jurkat cell line cultured for 24, 48 and 72 hours in the presence of various concentrations of Au/ZnO/Ag nanoparticles synthesized using *G. hederacea* L. extract

In this study, we used Au/ZnO/Ag nanoparticles against leukemia. The cytotoxicity of Au/ZnO/Ag nanoparticles was evaluated by means of the MTT assay [37, 38]. For this purpose, The analysis showed that the highest reduction in the relative cell viability (RCV) value occurred at the concentration range from 2 µmol to 6 µmol. The observed reduction was between 80% and 5% of live cells. The IC50 index was 2.63 µmol at 24 hours, 1.75 µmol at 48 hours and 1.24 µmol at 72 hours of culture. The total loss of cell viability was observed for the nanoparticle concentration range from 10 µmol to 50 µmol (Fig. 7).

## Annexin V binding test

**Fig. 8:**
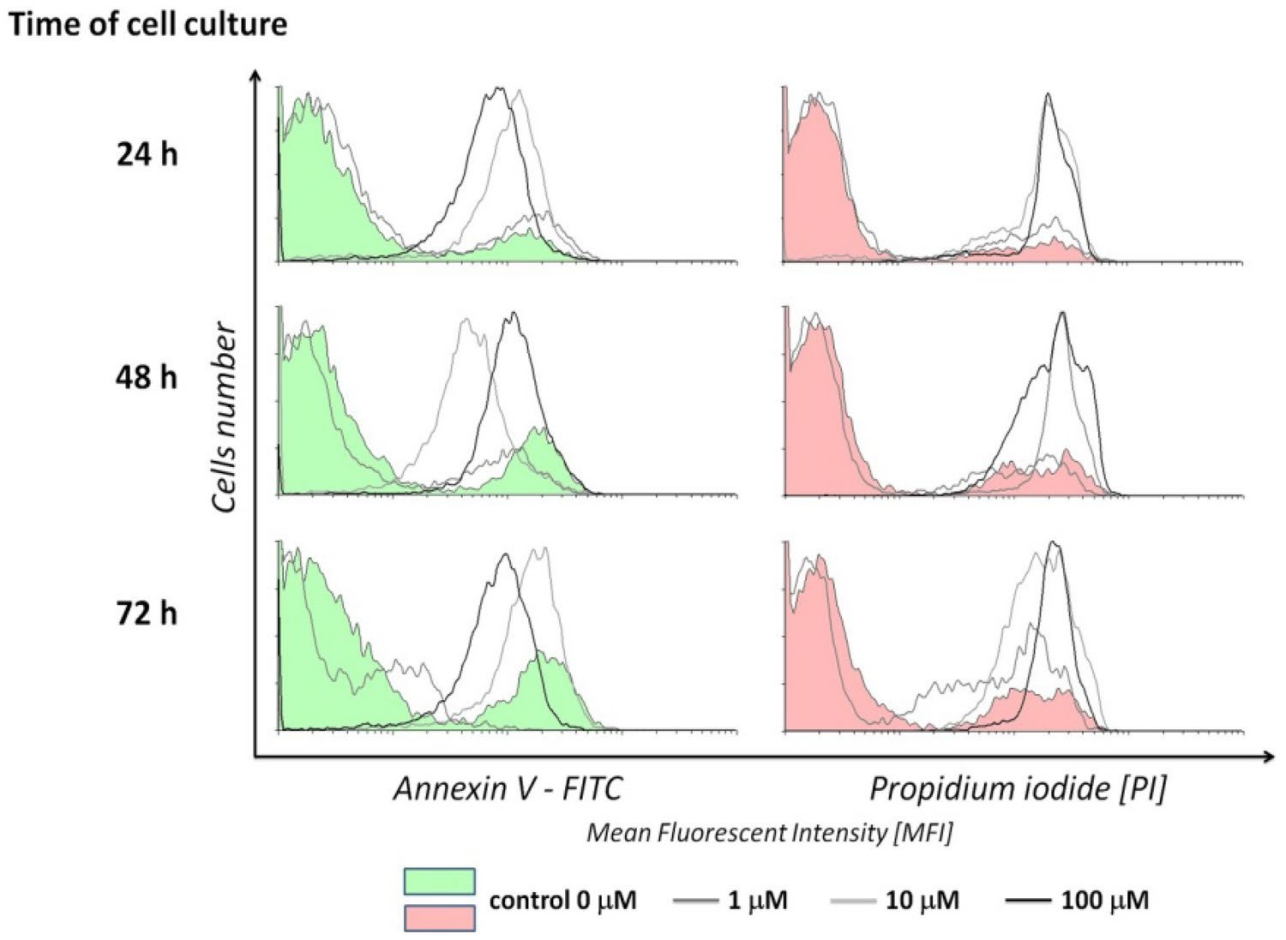
Mean Fluorescence Intensity of fluorescein-bonded Annexin V (FITC) and propidium iodide (PI) used for the staining of apoptotic and necrotic Jurkat cells cultured for 24, 48, and 72 h in the presence of various concentrations of Au/ZnO/Ag nanoparticles synthesized using *G. hederacea* L. extract

**Fig. 9:**
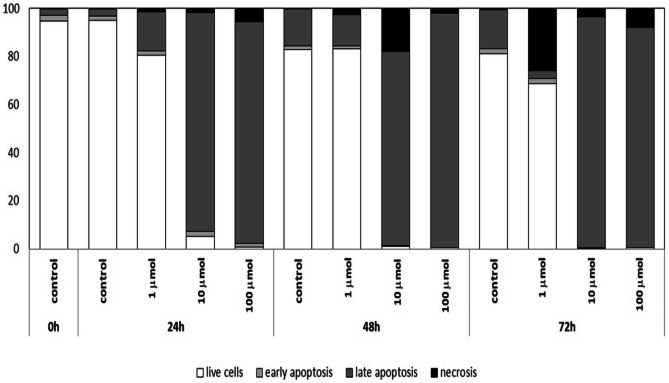
Percentages of Jurkat cells cultured for 24 and 48 hours in the presence of different concentrations of Au/ZnO/Ag nanoparticles synthesized using *G. hederacea* L. extract, showing a state of early apoptosis, late apoptosis and necrosis

The type of Jurkat cell death caused by Au/ZnO/Ag nanoparticles was identified by means of an apoptosis test, which detects cell membrane asymmetry. The test was carried out with the use of FITC-conjugated Annexin V, a Ca2+-dependent protein showing a strong affinity for phosphatidylserine residues externalized by apoptotic cells. Propidium iodide, a fluorochrome that penetrates the discontinuous cell membranes of necrotic cells, was used as the marker of necrosis (Fig. 8). The analysis demonstrated that nanoparticles caused the death of Jurkat cells. The intensity of changes was determined by the concentration of nanoparticles and cell culture time. At 24 hours of culture, Au/ZnO/Ag nanoparticles at a concentration of 10 µmol caused late apoptosis and necrosis in over 90% of Jurkat cells. Au/ZnO/Ag nanoparticles at the concentration of 100 µmol eliminated almost all living cells from the culture within 24h. This observation confirms the result of the MTT assay. The studied nanoparticles at the concentration of 1 µmol killed about 26% of Jurkat cells within 72h. Compared to the results obtained for control cells cultured without nanoparticles, it was an over 40-fold increase in necrosis. Throughout the study, control cells showed comparable necrosis maintained at 0.2-0.6% (Fig. 9).

## Cell cycle analysis

**Fig. 10:**
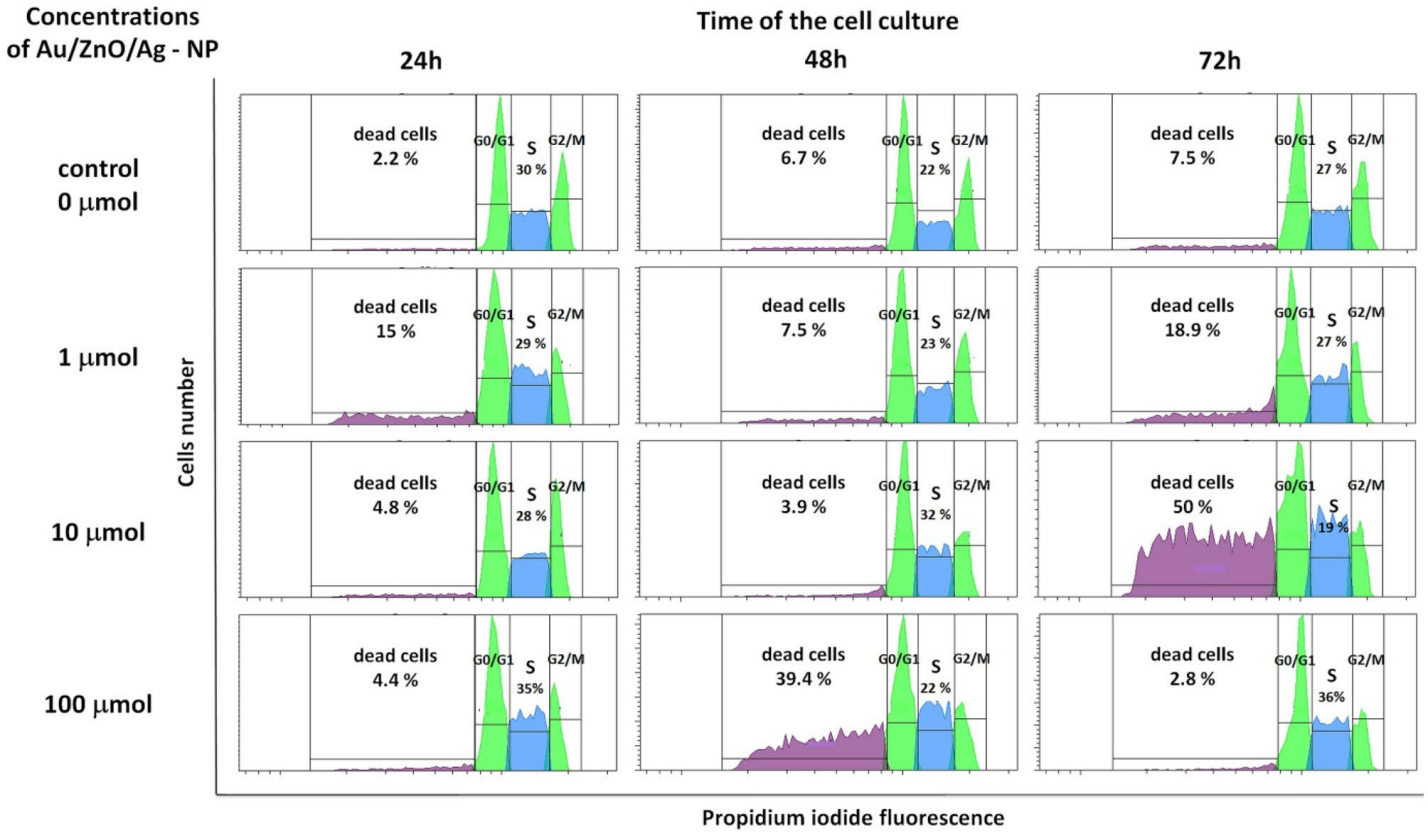
Analysis of the cell cycle of the Jurkat cell line cultured in various time periods in the presence of Au/ZnO/Ag nanoparticles synthesized using *G. hederacea* L. extract

**Fig. 11:**
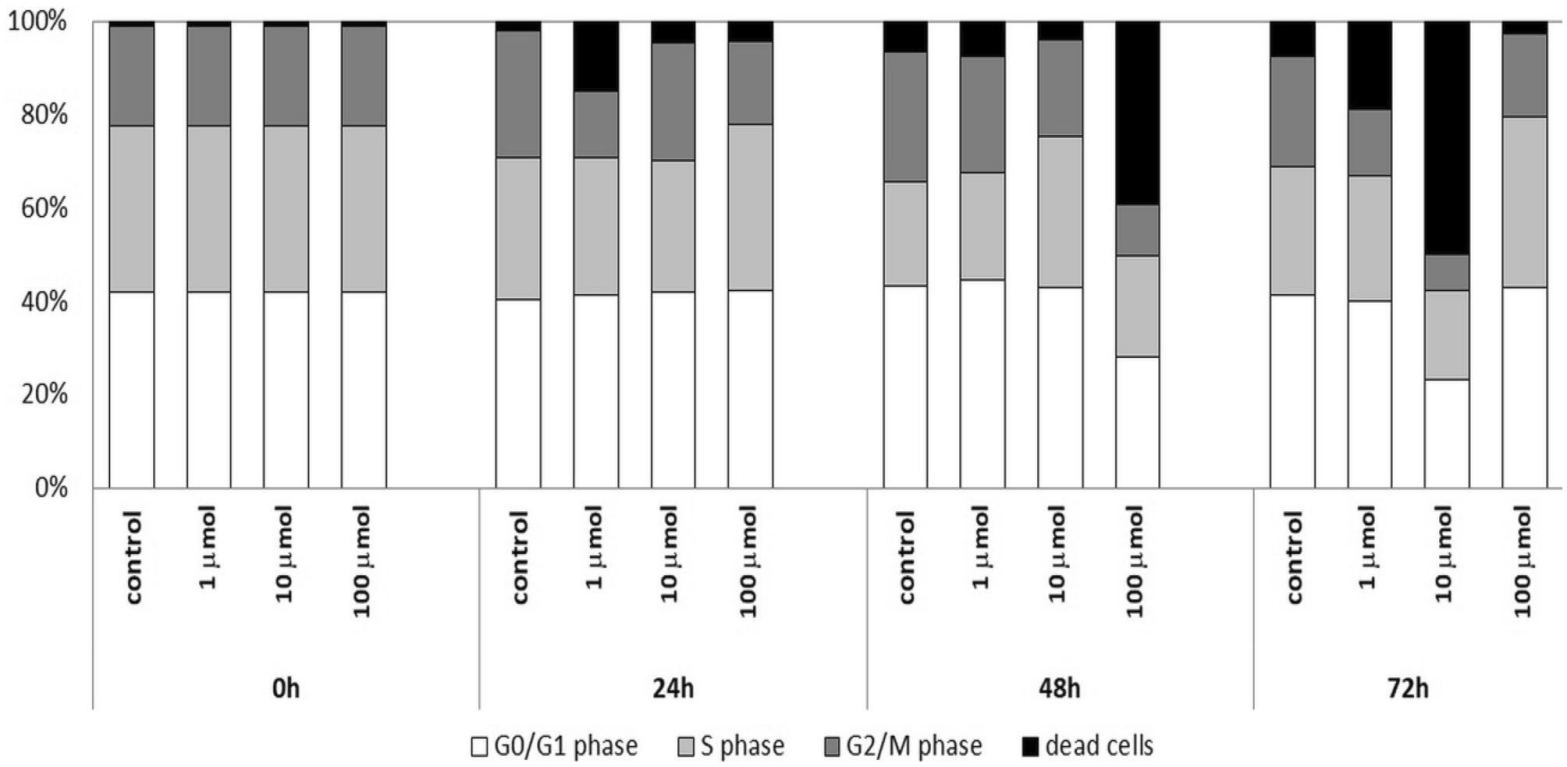
Percentages of Jurkat cells cultured for 24, 48, and 72 hours in the presence of various concentrations of Au/ZnO/Ag nanoparticles synthesized using *G. hederacea* L. extract remaining in G01/G1, S and G2/M phases of the cell cycle

**Fig. 12:**
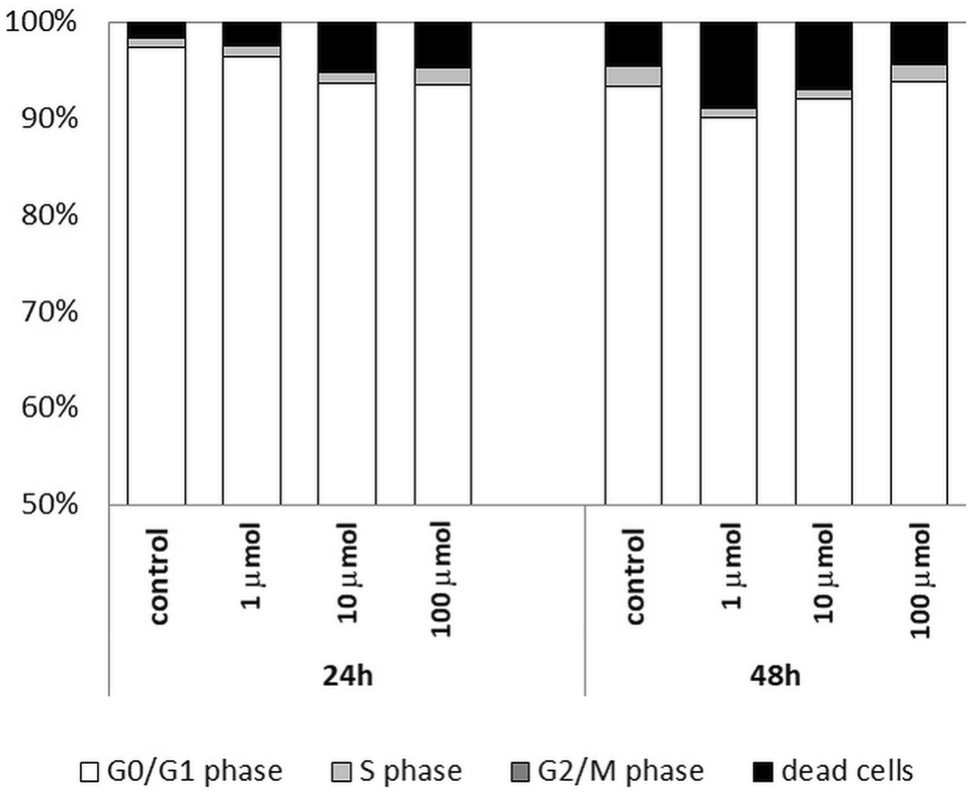
Percentages of peripheral blood lymphocytes of healthy donors cultured for 24 and 48 hours in the presence of different concentrations of Au/ZnO/Ag nanoparticles synthesized using *G. hederacea* L. extract remaining in G01/G1, S and G2/M phases of the cell cycle

The use of propidium iodide made it possible to assess the cell cycle of Jurkat cells cultured with 1 µmol, 10 µmol and 100 µmol of Au/ZnO/Ag nanoparticles. The analysis was conducted after 24, 48 and 72 hours of culture (Fig. 10). The results of the analysis were consistent with the MTT test and the Annexin V binding test, i.e. the percentage of dead cells increased with the time of culture and proportionally to the concentration of Au/ZnO/Ag nanoparticles. The addition of 1 μmol and 10 μmol of nanoparticles led to a systematic increase in the percentage of dead cells. The maximum result was obtained at 72h of culture. The addition of 100 μmol of Au/ZnO/Ag nanoparticles caused significant acceleration of this process; the maximum result was obtained at 48h of the test. Whenever the tested nanoparticles were used, massive cell death was preceded by inhibition of proliferative activity observed in the S phase of the cell cycle followed by an increase in its intensity (Fig. 11). Moreover, we used the test of analysis of variance (ANOVA) for values observed in the course of the Jurkat cell line cell cycle evaluation (Table 1). The cell cycle of peripheral blood mononuclear cells (PBMC) isolated from healthy donors’ whole peripheral blood was analyzed in order to show how the studied nanoparticles can potentially affect non-malignant healthy cells. Similarly as in the case of Jurkat cells, the number of dead PBMCs increased with culture time and the concentration of nanoparticles added. Table 2 shows the test of analysis of variance (ANOVA) for values observed in the course of the PBMC cell cycle evaluation. However, unlike Jurkat cells, PBMCs were much less sensitive to the tested nanoparticles; the percentage of dead cells was significantly lower, and it did not exceed 18% at 48 hours of culture (Fig. 12).

**Table 1:** The test of analysis of variance (ANOVA) for values observed in the course of the Jurkat cell line cell cycle evaluation

	sum of squares (SS)	degrees of freedom (df)	mean of squares (MS)	analysis of variance (F statistics)	test probability (*P* value)
G0/G1 phase					
24h	4.7	3	1.6	22.1	*P*<0.05
48h	360.7	3	120.2	3206	*P*<0.0001
72h	511.2	3	170.4	4544	*P*<0.0001
S phase					
24h	58.5	3	19.5	503,3	*P*<0.0001
48h	142.9	3	47.6	1411,0	*P*<0.0001
72h	295.0	3	98.3	2314,0	*P*<0.0001
G2/M phase					
24h	213.7	3	71.2	2590	*P*<0.0001
48h	327.6	3	109.2	7941	*P*<0.0001
72h	273.7	3	91.2	2433	*P*<0.0001
dead cells					
24h	198.9	3	66.3	2210	*P*<0.0001
48h	1665.0	3	554.9	8704	*P*<0.0001
72h	2712.0	3	903.9	24100	*P*<0.0001

**Table 2:** The test of analysis of variance (ANOVA) for values observed in the course of the PBMC cell cycle evaluation

	sum of squares (SS)	degrees of freedom (df)	mean of squares (MS)	analysis of variance (F statistics)	test probability (*P* value)
G0/G1 phase					
24h	46.91	3	15.64	7.079	*P*<0.01
48h	29.68	3	9.82	0.532	n.s.
S phase					
24h	0.95	3	0.32	0.654	n.s.
48h	3.67	3	1.22	1.018	n.s.
G2/M phase					
24h	-	-	-	-	-
48h	0.02	3	0,01	0.575	n.s.
dead cells					
24h	36.71	3	12.24	7.480	*P*<0.01
48h	54.01	3	18.00	1.088	n.s.

*n.s.* not significant

## Statistical analysis

Statistical analysis of the results obtained during the assessment of the cell cycle performed with the ANOVA with post-hoc Tukey's Multiple Comparison Test showed the statistical significance of the observed differences in the percentage of cells in the S and G2/M phases and dead cells between the tested cells treated with Au/ZnO/Ag-NPs and the control, regardless of the concentration and at selected time points. Statistical analysis of the results obtained during the Annexin V binding test performed with the ANOVA post-hoc Tukey's Multiple Comparison Test showed the statistical significance of the observed differences in the percentage of cells showing of early and late apoptosis as well as necrosis between cells treated with Au/ZnO/Ag-NPs and the control, regardless of concentration and at selected time points. Table 3 showes the test of analysis of variance (ANOVA) for values observed in the course of the Jurkat cell line Annexin V binding evaluation

**Table 3:** The test of analysis of variance (ANOVA) for values observed in the course of the Jurkat cell line Annexin V binding evaluation

	sum of squares (SS)	degrees of freedom (df)	mean of squares (MS)	analysis of variance (F statistics)	test probability (*P* value)
early apoptosis					
24h	0.094	3	0.031	0.019	n.s.
48h	0.650	3	0.217	0.463	n.s.
72h	1.455	3	0.485	0.483	n.s.
late apoptosis					
24h	3415	3	1138.0	0.538	n.s.
48h	2881	3	960.2	0.471	n.s.
72h	3591	3	1197.0	0.537	n.s.
necrosis					
24h	15.00	3	5.001	2.148	n.s.
48h	97.96	3	32.650	0.798	n.s.
72h	192.90	3	64.310	0.691	n.s.

*n.s.* not significant

## Conclusion

The interdisciplinary study of nanoscience and biology is a gateway for the development and synthesis of novel nanomaterials in different fields of science. Current oncological research has shown that there are numerous cancer therapy modalities that could highly benefit from the recently developed nanomaterials, including metal nanoparticles. The biological synthesis of metal nanoparticles has gained the researchers’ interest as a possible environmentally-friendly substitute for physical and chemical approaches, which are expensive and hazardous. In this work, *G. hederacea* L. extract was used to carry out the biological synthesis of Au/ZnO/Ag nanoparticles. The obtained spherical Au/ZnO/Ag nanoparticles of about 50–70 nm in size were evaluated in terms of their cytotoxic activity against leukemia. The studies showed that the total loss of cell viability occurred at the Au/ZnO/Ag nanoparticle concentration range of 10 µmol–50 µmol. Additionally, Au/ZnO/Ag nanoparticles at the concentration of 100 µmol eliminated almost all living cells from the culture within 24h. This observation confirms the result of the MTT test. The number of dead PBMCs increased with culture time and the concentration of added nanoparticles. However, unlike Jurkat cells, PBMCs were significantly less sensitive to the presence of the tested nanoparticles; the percentage of dead cells was much lower and did not exceed 18% at 48 hours of culture.
